# Editorial

**Published:** 1865-10

**Authors:** 


### Editorial.

Medical Colleges.—The general introductory lecture to the annual course of instruction in the Chicago Medical College, Medical Department of Lake Forest University, was given in the lecture-room of the College on the evening of the 2d inst. The exercises were opened with prayer by the Rev. J. H. Vincent, and the introductory lecture delivered by J. H. Hollister, M.D., Professor of Physiology and Histology. The lecture was elegantly written, and in sentiment well calculated to interest and profit those to whom it was addressed.

The number of students in attendance at the opening of the term is, at least, one-third larger than in any previous year; and the prospects are good for full courses of instruction in every department of medical science and art.

The chemical laboratory is well-furnished; several important additions have been made to the museum, more especially for the purpose of illustrating the courses on anatomy, physiology, microscopy, and surgery: and the wards of Mercy Hospital are crowded with patients, furnishing an ample field for clinical instruction. The full five months' lecture-term, with a supplementary summer reading and clinical-term of four months; the arrangement for enabling the student to make his courses progressive instead of repetitional; and the full daily course of true clinical instruction in medicine aud surgery, are advantages that are rapidly becoming known and appreciated by the profession.

The general introductory lecture in the Rush Medical College was given on Wednesday evening, the 4th inst., by Prof. J. V. Z. BLANEY, who having terminated his service in the medical department of the army, resumes the duties of Professor of Chemistry, as formerly. His lecture was well written and well received by the class.

The number of students in attendance was large, and the College represented to be in a prosperous condition. Its greatest defects are the shortness of the lecture-term, and the very limited advantages for clinical instruction. For though the Professors of Surgery and Practical Medicine have each appended to their titles that of "Clinical Professor," yet neither has a place in any hospital in the city, or any means for clinical instruction, except the simple college dispensary.

---

MEDICAL COLLEGES IN CINCINNATI.—Since the death of Dr. A. H. BAKER, the faculty of the Cincinnati College of Medicine and Surgery has been reorganized, with Dr. WOOD in the Chair of Surgery.

*The Miami Medical College* has abolished lectures altogether, and in their place demand simply a matriculation fee of $15.

*Berkshire Medical College.*—Dr. HORATIO R. STORER, of Boston, has been appointed to the Chair of Obstetrics in this institution.

---

EXPLANATION.—The article on "Beebread as a Diuretic," in the September number of the EXAMINER, and the one on "Forcible Dilatation of Strictures of the Urethra," in the present number, were written in 1859, and sent to me about the time that I transferred the *Chicago Medical Journal* to Dr. D. BRAINARD. They were accidentally placed among some papers where they were not discovered again until a few weeks since. We have chosen to give them publicity at this late period, because the subjects to which they relate are still of practical interest to the profession. That relating to strictures has no name attached to it, but it was doubtless written by Dr. Edwin Powell, of this city, who was, at that time, one of the surgeons to the Mercy Hospital.

---

The Mortatity Statistics for August.—The Mortality report of the City of Chicago, for the month ending Aug. 31, has been prepared by the health officer, T. B. Bridges. The entire number of deaths was 464, being a decrease of 35 over the corresponding month of last year, notwithstanding the great increase in the population.

DISEASES.

| | | | |
|---|---|---|---|
| Apoplexy, | 2 | Inflammation of Lungs, | 7 |
| Accident, | 15 | Inflammation of Stomach, | 1 |
| Brain Fever, | 5 | Jaundice, | 1 |
| Bilious Fever, | 2 | Liver Complaint, | 1 |
| Burned, | 1 | Lung Fever, | 3 |
| Congestion of Brain, | 4 | Measles, | 1 |
| Congestion of Lungs, | 2 | Marasmus, | 4 |
| Congestion of Liver, | 1 | Nervous Fever, | 3 |
| Congestive Chills, | 1 | Old Age, | 7 |
| Cholera Morbus, | 3 | Paralysis, | 1 |
| Cholera Infantum, | 14 | Sunstroke, | 1 |
| Cold, | 1 | Scalded, | 1 |
| Cancer, | 1 | Shot by Police, | 2 |
| Consumption, | 31 | Suppressed Menstruation | 1 |
| Cramps, | 12 | Suicide, | 3 |
| Croup, | 8 | Spotted Fever, | 1 |
| Childbirth, | 2 | Scarlet Fever, | 11 |
| Convulsions, | 5 | Summer Complaint, | 114 |
| Drowned, | 6 | Stillborn, | 12 |
| Diphtheria, | 9 | Spasms, | 2 |
| Dropsy, | 11 | Small-Pox, | 1 |
| Dysentery, | 25 | Teething, | 22 |
| Diarrhœa, | 22 | Typhoid Fever, | 13 |
| Heart Disease, | 3 | Unknown, | 39 |
| Hemorrhage, | 3 | Whooping Cough, | 3 |
| Intermittent Fever, | 2 | | |
| Inflammation of Brain, | 1 | Total, | 464 |
| Inflammation of Bowels, | 8 | | |

Ages of the Deceased.—Under 5 years, 312; over 5 and under 10 years, 12; over 10 and under 20, 25; over 20 and under 30, 32; over 30 and under 40 42; over 40 and under 50, 17; over 50 and under 60, 9; over 60 and under 70 6; over 70 and under 80, 5; unknown, 4. Total, 464.

NATIVITIES.

| | | | | | |
|---|---|---|---|---|---|
| Chicago, | 289 | England, | 4 | Sweden, | 3 |
| Other States, | 53 | Germany, | 32 | Wales, | 2 |
| At Sea, | 1 | Ireland, | 48 | Unknown, | 10 |
| Bohemia, | 3 | Italy, | 1 | | |
| Canada, | 7 | Norway, | 7 | Total, | 464 |
| Denmark, | 3 | Scotland, | 1 | | |

DIVISIONS OF THE CITY.

| | | | | | | | |
|---|---|---|---|---|---|---|---|
| North, | 127 | South, | 139 | West, | 198 | Total, | 464 |

Appended is the official mortality report for the month of September, 1865, as taken from the records of the health officer. It will be seen that the statistics, as compared with the month preceding, show a large decrease of the number of deaths, due, perhaps, mainly to the cooler weather experienced during the latter part of the month. But a feature not less gratifying is the fact that, with a population largely increased over that of 1864, the deaths for September of this year show a falling off of 48, as compared with the same month last year. This is doubtless to be attributed in large measure to a better system of sanitary precautions, and to the fact that some miles of sloughs and filthy streets have within the past year been covered from sight and smell with the pavements which have been put down in various sections:—

DISEASES.

| | | | |
|---|---|---|---|
| Apoplexy, | 1 | Inflammation of Lungs, | 2 |
| Accidents, | 11 | Intemperance, | 2 |
| Brain Fever, | 4 | Jaundice, | 1 |
| Billious Fever, | 6 | Liver Complaint, | 1 |
| Congestion of Brain, | 3 | Lockjaw, | 1 |
| Cholera Morbus, | 6 | Nervous Fever, | 3 |
| Cholera Infantum, | 4 | Old Age, | 7 |
| Cancer, | 1 | Sunstroke, | 1 |
| Cerebro-Spinal Meningitis, | 1 | Ship Fever, | 1 |
| Consumption, | 31 | Scarlet Fever, | 12 |
| Cramps, | 18 | Summer Complaint, | 54 |
| Croup, | 14 | Stillborn, | 6 |
| Childbirth, | 5 | Spasms, | 1 |
| Convulsions, | 13 | Small-Pox, | 1 |
| Drowned, | 4 | Teething, | 16 |
| Diphtheria, | 11 | Typhoid Fever, | 14 |
| Dropsy, | 11 | Typhus, " | 2 |
| Dysentery, | 19 | Tabes Mesenterica, | 1 |
| Diarrhœa, | 9 | Whooping Cough, | 2 |
| Heart Disease, | 2 | Worms, | 1 |
| Intermittent Fever, | 5 | Unknown, | 31 |
| Inflammation of Brain, | 3 | | —— |
| " " Bowels, | 4 | Total, | 346 |

Ages of the Deceased.—Under 5 years, 189; over 5 and under 10 years 24; over 10 and under 20, 12; over 20, and under 30, 28; over 30 and under 40, 40; over 40 and under 50, 17; over 50 and under 60, 14; over 60 and under 70, 12; over 70 and under 80, 6; over 80 and under 90, 1; unknown, 3. Total, 346.

NATIVITIES.

| | | | | | |
|---|---|---|---|---|---|
| Chicago, | 193 | England, | 4 | Sweden, | 9 |
| Other States, | 41 | Germany, | 38 | Wales, | 1 |
| Bohemia, | 3 | Ireland, | 40 | Unknown, | 4 |
| Canada, | 2 | Norway, | 6 | | — |
| Denmark, | 2 | Scotland, | 3 | Total, | 346 |

DIVISIONS OF THE CITY.

North, 94 | South, 98 | West, 154 | Total, 346

